# Comparative study of postpartum sexual function: Second-degree tears versus episiotomy outcomes

**DOI:** 10.1007/s00404-024-07494-2

**Published:** 2024-04-13

**Authors:** María José Fernández-Fernández, Alejandro Jesús de Medina-Moragas

**Affiliations:** 1 Andalusian Health Service, Seville, Spain; 2 Faculty of Nursing, Physiotherapy and Podiatry, University of Seville, Seville, Spain

**Keywords:** Dyspareunia, Tearing, Episiotomy, Sexual Health, Perineal Trauma

## Abstract

**Purpose:**

The effects of episiotomy and second-degree tears on postpartum sexual function are key areas of enquiry in women’s health research. Episiotomy and second-degree tears are common procedures and injuries that occur during childbirth. Understanding their impact on post-childbirth sexuality is crucial to women’s overall well-being. This study aimed to examine the relationship between episiotomy, second-degree tears, and post childbirth sexuality.

**Methods:**

A cross-sectional design was employed, including 83 women who gave birth to Cáceres in 2017. Participants were evaluated based on sociodemographic and sexual health factors.

**Results:**

No significant differences were found in dyspareunia or sexual function between women who underwent episiotomies and those with second-degree tears. However, women who underwent episiotomies waited longer before resuming sexual activity after childbirth. Factors such as age, number of previous births, employment status, educational level, and breastfeeding status affected the timing and frequency of postpartum sexual activity.

**Conclusion:**

Dyspareunia negatively affects various aspects of sexual function. When comparing episiotomy and second-degree tears, their impacts on postpartum sexual function were similar. However, episiotomy delays the resumption of sexual activity. Sociodemographic factors significantly influence postpartum sexual health. These findings highlight the importance of individualised interventions and support for new mothers during the postpartum period to address potential sexual health concerns.

## Take-home message:

**Table:** 

This study compares episiotomies and second-degree perineal tears on postpartum sexual function, focusing on the delay in resuming sexual intercourse and its impact on sexual health. There were no major differences in sexual health dimensions between women with episiotomy and second-degree tear, except for a longer resumption of sexual activity after episiotomy.	

## Introduction

Sexuality is a complex aspect of overall well-being encompassing physical, mental, emotional, and social dimensions. Extensive research has been conducted on this topic by experts such as Master and Johnson, who conducted a notable study involving 100 couples [1]. One notable advancement is Basson’s theoretical framework, which outlines four distinct phases of sexual responses: arousal, plateau, orgasm, and resolution. It is important to acknowledge that female sexual function is influenced by relational dynamics, with instances in which arousal precedes the desire for sexual engagement because of the emphasis on emotional intimacy. Nonlinear models, such as Basson’s cyclic biopsychosocial model, consider interpersonal relationships, emotional intimacy, and other psychosocial factors to provide a more comprehensive view [2, 3]. The postpartum period, characterized by the transformative experience of childbirth, brings about various challenges and transformations in couples’ sexual lives. It is widely studied that many women experience a decline in their sexual health following childbirth, with decreased sexual frequency, lower libido, and reduced pleasure during pregnancy and the postpartum period. Research supports the influence of childbirth on sexual dynamics among partners, indicating that multiple factors contribute to these shifts [4]. This complex phenomenon is influenced by various sociodemographic variables that affect postpartum sexual health and childbirth experience. This may increase the risk of perineal tears or episiotomies [5]. Furthermore, breastfeeding can affect sexual function after childbirth. Hormonal changes that occur during breastfeeding can affect libido and vaginal lubrication [6]. Multiple childbirths can have an impact on postpartum sexual health as they may result in more elastic perineal tissues and decrease the risk of severe tears. However, multiple childbirths can also increase the risk of complications related to prior tears or episiotomy [5]. An important aspect to consider for understanding postpartum sexual health and the occurrence of tears or episiotomies is Body Mass Index, newborn weight, and the time elapsed before resuming sexual activity. Higher BMI levels may contribute to more difficult childbirths and an increased risk of tears, whereas heavier babies can pose higher risks [7]. The length of time that couples abstain from sexual activity after childbirth can have a significant impact on the level of pain or discomfort, especially in cases with tears or episiotomies [8]. To foster empathy and communication in relationships, it is important to comprehensively understand the common challenges faced by couples after childbirth [9, 10].

After childbirth, many women experience dyspareunia, which is characterized by genital pain during or after sexual intercourse. Studies have shown that the prevalence of dyspareunia at 12 weeks postpartum ranges from 11.9% to 31.4% [11]. Furthermore, it is frequently reported that women experience pain during penetration two months after giving birth [12]. Dyspareunia is a common issue that arises in the first to 3–6 months after childbirth, leading to a delay in resuming sexual activity and impacting women’s overall quality of life. This condition is often caused by obstetric factors, such as perineal tears or episiotomies during vaginal delivery. Extensive research has explored various risk factors, including birth type and breastfeeding status. Perineal tissue damage significantly affects the sexual lives of women, particularly dyspareunia. Women with an intact perineum tend to resume sexual activity sooner than those who have experienced perineal trauma such as tears or episiotomy [13]. Significantly, a notable percentage of women with severe perineal injuries (75%) and moderate injuries (61.8%) continued to experience perineal pain three months after childbirth. By the six-month mark, perineal pain persisted in 60% and 38.7% of women with severe and moderate injuries, respectively. While the prevalence of perineal pain decreases over time, 23% of women still experience pain one year after giving birth [13].

Perineal injuries resulting from childbirth are a significant concern because of their potential long-term consequences on women’s health. These injuries, which range in severity from mild to severe, can have lasting implications. Grade 1 tears only affect the perineal skin, while grade 2 tears involve both the muscles and skin. More severe tears, grades 3 and 4, extend towards the anal sphincter complex or rectal mucosa, respectively. Typically, midwives handle the repair of grade 2 tears, while gynecologists address grade 3 and 4 tears. Dyspareunia commonly occurs after perineal tearing, particularly in high-grade tears. Second-degree vaginal trauma during childbirth increases the risk of dyspareunia. Episiotomy, a surgical procedure that enlarges the vaginal opening through an incision in the perineum, can have similar effects and negatively impacts postpartum sexual well-being. Although episiotomies are now less commonly performed due to concerns about increased blood loss and complications, they may still be recommended in certain situations, such as fetal distress or instrumental vaginal birth. Several studies have found a link between episiotomy and dyspareunia six months after delivery. Women who do not undergo episiotomies engage in sexual activity more frequently, and experience dyspareunia less often [13, 14]. Moreover, a considerable proportion of women who experienced dyspareunia three months after giving birth underwent episiotomy [15, 16]. Limited research has been conducted on the effect of perineal tears on sexual function. However, it is important to note that perineal trauma can have a negative impact on sexual function, including decreased libido, reduced frequency of orgasms, diminished satisfaction with sex, and increased pain during intercourse [17, 18]. Although many studies have explored the prevalence of dyspareunia after childbirth, few have examined its potential risk factors. The relationship between dyspareunia, timing of sexual function recovery postpartum, and perineal trauma during labor is still not well understood [15, 19]. Therefore, although previous research has indicated a connection between postpartum sexual dysfunction, specifically dyspareunia, and perineal trauma caused by episiotomy or second-degree tears during childbirth, no study has specifically examined this relationship in severe cases.

Existing research on the prevalence and causes of postpartum dyspareunia is available; however, few studies have directly compared the effects of episiotomies and second-degree perineal tears on postpartum sexual function. This study, with its distinct focus on comparing postpartum sexual outcomes between women with episiotomies and those with second-degree perineal tears, seeks to address this gap in understanding. By shedding light on the specific effects of these two common obstetric procedures on postpartum sexual health, this study aims to provide valuable insights for medical interventions during childbirth and offer essential guidance for postpartum counselling, ensuring that women are well-informed about potential sexual health issues after giving birth.

The primary objective of this study was to investigate the effect of episiotomy and second-degree perineal tears on postpartum sexual function in women attending the Cáceres Birth Room. Furthermore, the study aimed to compare the consequences of second-degree perineal tears and episiotomy on sexual function within the same cohort of women three months postpartum. It was hypothesized that episiotomy negatively affects sexual function at three months postpartum in women attending the Cáceres Birth Room compared to second-degree perineal tears.

## Methods

This study employed a cross-sectional design and adhered to the guidelines outlined in the STROBE statement, a highly regarded framework that provides explicit criteria to ensure the optimal quality of observational studies [20]. The recruitment process for this study was implemented throughout the entire year of 2017, from January 1st to December 31st, at San Pedro de Alcántara Hospital. All women who gave birth at the hospital during this period and fulfilled the inclusion criteria were systematically approached to participate in the study, thereby ensuring a comprehensive and representative sample of the postpartum population. Eligible participants were contacted by phone 12 weeks after giving birth, and contact information was obtained from the birth room and electronic medical records. Participants were provided with clear instructions and had the opportunity to address any doubts, ensuring their full comprehension of the study’s purpose and procedures, thereby enhancing data quality. The study population consisted of women who had given birth at the San Pedro de Alcántara Hospital in Cáceres in 2017. These women were attended by midwives and had undergone euthyroid births after 37 weeks of gestation with specific conditions such as second-degree tears or mid-lateral episiotomies. This selection criterion was employed to ensure homogeneity in birth conditions and to focus on a particular group of women who experienced these specific conditions during childbirth. Several exclusion criteria were applied to maintain the internal validity of the study. Women with first-, third-, or fourth-degree tears; those reporting dyspareunia prior to their latest delivery; those with a history of prior perineal surgery; and cases involving fetal death, extensive congenital anomalies, severe condylomas, extensive vulvar varicose veins, lower genital tract pathology, and instances of episiotomy with tears were excluded from the study. Since no reference studies were available to determine the ideal sample size for this specific research question, a non-probability convenience sample was deemed suitable. A threshold of more than 30 participants is generally considered adequate in statistical practice to yield robust and reliable results. Consequently, the study included 39 women with tearing and 44 women who had undergone episiotomies in their cohort. Prior to participating, all individuals provided informed consent and fully understood the study’s objectives, significance, assurance of confidentiality, and potential advantages. These ethical precautions were taken to protect the rights and privacy of the participants and maintain the overall integrity of the study.

The sociodemographic characteristics of interest in this study included age, country of birth, marital status, educational level, and employment status. These variables were selected because of their potential influence on postpartum experiences and recovery. A dedicated form was meticulously designed to collect relevant information pertaining to these variables systematically. To be eligible for inclusion in the study, participants had to be at least 16 years old, as younger women might have had additional risk factors or different childbirth experiences. The following variables were assessed in this study and collected using a questionnaire: breastfeeding, number of births, body mass index (BMI), newborn birth weight, and time of resumption of sexual relations after childbirth. This study focused on second-degree tears, as these are the highest degree of tears typically treated by midwives in Spain. This ensured that the research was centered on the most common and relevant injuries managed by midwives in this context and data on this variable were obtained from birth registries. To assess sexual function in women, a questionnaire on women’s sexual function (WSF) was administered. This self-administered questionnaire consisted of 14 closed questions and one alternative, utilizing a 5-value Likert scale and integrating them into domains. The questionnaire covered essential aspects, such as sexual frequency, the presence of a partner, and factors crucial for diagnosing sexual dysfunction, including dyspareunia. This validated questionnaire assessed various phases of sexual response, initiative, and degree of sexual communication. It collected descriptive data on sexual performance and proved valuable in exploring and diagnosing sexual dysfunction. Notably, this questionnaire has demonstrated reliability and content validity in the Spanish population. A Cronbach’s *α* value of 0.93 was obtained in the present study, aligning with the stability and validity reported by Sánchez et al. who found a Cronbach’s *α* value of 0.89 [21]. Pain during sexual activity was evaluated as a discrete quantitative variable using a visual analogue scale (VAS) ranging from 0 to 10. This psychometric instrument was employed to quantify the subjective characteristics that can be challenging to measure using numerical scales. The participants marked a point on a straight line with categorized endpoints to signify their perception of pain during sexual activity [22]. Descriptive statistics, including frequency tables and key statistics, such as mean and standard deviation, were employed to identify and rectify any errors related to data transcription and coding on the data collection sheet. Subsequently, the study’s sample of participants is described, summarizing their primary characteristics to ensure data accuracy. Correlations were established using Spearman’s correlation diagrams, and assumptions of linearity and normality were verified. Effect sizes were categorized as small (0.10–0.30), moderate (0.30–0.50), or large (above 0.50) [23]. Statistical analysis was conducted using the Mann–Whitney U test to assess differences in sexual function postpartum between women with second-degree tears and episiotomy. This test was chosen because of the non-normal distribution of data. The test evaluated various indicators of sexual function, and p-values less than 0.05 are highlighted in bold, indicating statistical significance. Although these findings were statistically robust, they were also considered within a broader clinical context to determine their practical significance in postpartum care. It is important to note that no changes can be made to the citation, reference, or in-line citations and that the numbers in the text should not be modified.

Multivariate regression models incorporating statistically significant variables were refined using a backward stepwise regression. This process involved excluding highly correlated predictors to avoid collinearity and identifying confounders that influenced the β coefficients by 20% or more. The validity of the model was confirmed through model validation, which included checking for linearity, homoscedasticity, normality, and non-collinearity. In addition, a residual analysis was conducted to ensure compliance with the assumptions. The validity of the model was confirmed using the Snedecor F-statistic, and the null hypothesis was rejected at *p* < 0.05. All data analyses were conducted using specialized software.

## Results

The study included 83 female participants, with 39 having experienced spontaneous tears during childbirth and 44 who underwent an episiotomy. These participants hailed from diverse backgrounds, representing a total of nine countries: Spain (91.6%), England, Colombia, Uruguay, Paraguay, Germany, Honduras, and France. Regarding marital status, the majority of participants (72.3%) were married, 13.3% were engaged, 12% were single, and 2.4% were divorced. In terms of educational background, the highest proportion of participants (72.3%) had completed post-secondary education, followed by those with secondary education (20.5%) and a smaller group with primary education (7.2%). With regard to employment status, 42.2% of the participants were employed full-time, 34.9% were unemployed, and 22.9% worked part-time. Approximately 67% of women in the study were breastfed.

Table 1 provides the descriptive statistics for the quantitative variables. The average age of participants was 33.08 years, with a standard deviation of 4.63. On average, these women took approximately 45.78 days (with a standard deviation of 17.86) to initiate sexual relations after childbirth. In terms of sexual initiative, the mean score indicated a moderate level of sexual disorder among female participants. Our analysis of statistical data demonstrated substantial variations in certain postpartum sexual function parameters when comparing women with second-degree perineal tears to those who underwent episiotomy. As shown in Table 1, there were statistically significant differences in the age of the women (*p* = 0.02) and the number of childbirths (*p* = 0.00), with the mean age being higher and the number of births being greater in the second-degree tear group than in the episiotomy group. Furthermore, the initiation of sexual intercourse postpartum differed significantly between the groups (*p* = 0.022), with a delayed onset in the episiotomy group. However, other sexual function variables such as desire, excitement, lubrication, orgasm, and overall sexual satisfaction did not reveal statistically significant differences between the two groups (*p* > 0.05).

**Table 1:** Comparative analysis of postpartum sexual function variables between second-degree tear and episiotomy groups using Mann–Whitney U-test

	Second-degree tear		Episiotomy		Total sample		*p*-value
	M	SD	M	SD	M	SD	
Age	34.23	4.94	32.07	4.13	33.08	4.63	**.02**
Number of births	1.90	.59	1.41	.58	1.64	.63	**.00**
Woman height	1.63	.07	1.63	.05	1.63	.06	.57
Woman’s weight	66.76	13.32	63.79	10.27	65.19	11.82	.45
Newborn weight	3.28	.46	3.29	.60	3.28	.53	.94
Beginning of sexual intercourse	39.45	13.23	50.45	19.48	45.78	17.86	**.02**
Desire	9.32	2.08	9.44	2.30	9.39	2.19	.94
Excitement	10.74	3.03	10.64	2.90	10.69	2.94	.88
Lubrication	3.45	1.31	3.49	1.33	3.47	1.31	.85
Orgasm	3.94	1.41	3.97	1.28	3.96	1.33	.88
No problems with vaginal penetration	10.81	3.56	11.77	2.76	11.34	3.15	.33
Absence of anticipatory anxiety	3.67	1.37	3.71	1.20	3.69	1.27	.94
Sexual initiative	2.39	.88	2.74	1.11	2.59	1.02	.17
Degree of sexual communication	4.45	.81	4.46	.94	4.46	.87	.64
Satisfaction with sexual activity	8.13	2.47	8.28	2.13	8.21	2.27	.83
Overall sexual satisfaction	3.55	1.38	3.90	.96	3.74	1.17	.37
Frequency of sexual activity	1.97	1.04	1.85	.87	1.90	.95	.77

*M* mean, *SD* standard deviation; Statistical significance was determined using the Mann–Whitney: *p*-values < .05 is indicated in bold

The distribution of the tearing/episiotomy variable did not adhere to the normality assumptions based on various normality tests, including the Kolmogorov–Smirnov test with a significance level below 0.05. Consequently, nonparametric statistical tests were employed for bivariate correlation analysis using the Spearman rank correlation coefficient, and for hypothesis testing using the Kruskal–Wallis test. The analysis revealed several noteworthy correlations between the variables. Women’s age exhibited a low positive correlation with the number of births (rho = 0.33) and an inverse relationship with the frequency of sexual activity in the last four weeks (rho = −0.34). A higher level of partner commitment (e.g., married vs. engaged or unmarried) was weakly associated with a later onset of sexual intercourse after childbirth (rho = 0.31). Higher education levels were positively correlated with employment (rho = 0.29) and the later onset of sexual activity after childbirth (rho = 0.24). Conversely, education was negatively correlated with the frequency of sexual activity (*ρ* = −0.27). The correlation between employment and later initiation of sexual intercourse was weak (*p* = 0.33). Breastfeeding was positively correlated with higher educational attainment (rho = 0.32), greater birth weight of the newborn at birth (rho = 0.29), and a lower frequency of sexual activity (rho = −0.30). Female height was slightly positively correlated with the later onset of sexual activity (rho = 0.23) and newborn birth weight (rho = 0.21). Greater birth weight, in turn, was related to a lower incidence of dyspareunia (rho = −0.29). A later initiation of sexual intercourse was weakly correlated with a lower frequency of sexual activity (rho = −0.31). Vaginal penetration in the previous month was associated with fewer difficulties related to vaginal penetration (rho = 0.31).

Table 2 highlights statistically significant moderate or high correlations, indicating substantial relationships between the various dimensions of the WSF Sexual Health Questionnaire. Specifically, there were strong positive correlations between desire and arousal (rho = 0.71), which were, in turn, related to lubrication (rho = 0.88), orgasm (rho = 0.63), and satisfaction with sexual activity (rho = 0.66). Additionally, a high correlation was observed between orgasm and satisfaction with sexual activity (rho = 0.94) as well as between anticipatory anxiety and problems with vaginal penetration (rho = 0.77). These findings underscore the interconnected nature of these dimensions in the questionnaire and their influence on women’s sexual health and experiences.

**Table 2:** Spearman correlation coefficients (Rho)

	D	E	L	O	VP	AA	SI	SC	SA	SS	FS
Desire	1	**.71**	**.51**	**.51**	.33	.36	**.47**	.08	**.54**	.26	**.47**
Excitement	**.71**	1	**.88**	**.63**	**.49**	**.46**	**.44**	.25	**.66**	**.40**	**.40**
Lubrication	**.51**	**.88**	1	**.57**	**.55**	**.56**	.39	.21	**.58**	.38	.33
Orgasm	**.51**	**.63**	**.57**	1	**.42**	**.40**	.24	.27	**.94**	**.47**	.36
No problems with vaginal penetration	.33	**.49**	**.55**	**.42**	1	**.77**	**.42**	.25	**.51**	**.49**	.19
Absence of anticipatory anxiety	.36	**.46**	**.56**	**.40**	**.77**	1	**.46**	.20	**.45**	**.43**	.34
Sexual initiative	**.47**	**.44**	.39	.24	**.42**	**.46**	1	.16	.30	.28	.24
Degree of sexual communication	.08	.25	.21	.27	.25	.20	.16	1	.30	.23	.02
Satisfaction with sexual activity	**.54**	**.66**	**.58**	**.94**	**.51**	**.45**	.30	.30	1	**.51**	.33
Overall sexual satisfaction	.26	**.40**	.38	**.47**	**.49**	**.43**	.28	.23	**.51**	1	.11
Frequency of sexual activity	**.47**	**.40**	.33	.36	.19	.34	.24	.02	.33	.11	1

Bold scores mean: *p* < .05

*D* Desire, *E* Excitement, *L* Lubrication, *O* Orgasm, *VP* No problems with vaginal penetration, *AA* Absence of anticipatory anxiety, *SI* Sexual initiative, *SC* Degree of sexual communication, *SA* Satisfaction with sexual activity, *SS* Overall sexual satisfaction, *FS* Frequency of sexual activity

Regarding hypothesis testing, the nonparametric contrast test revealed no significant differences between the groups of women with tearing and those who underwent episiotomy in any of the dimensions of sexual health (Table 3). However, a statistically significant difference (*χ*^2^ = 5.28; significance level = 0.02) was found between the two groups in the initiation of sexual intercourse, with women who had tears taking an average of 40.22 (SD = 2.68) days to initiate sexual activity after childbirth, compared to women undergoing episiotomy, who took an average of 50.89 (SD = 3.23) days.

**Table 3:** Kruskal Wallis test results with the Tear/Episiotomy grouping variable

	DP	D	E	L	O	VP	AA	SI	SC	SA	SS	FS
χ2	1.35	.00	.02	.03	.02	.93	.00	1.85	.21	.04	.78	.08
*p*	.24	.94	.88	.85	.88	.33	.94	.17	.64	.83	.37	.77

*Χ*^2^: Chi-square

*DP* Dyspareunia, *D* Desire, *E* Excitement, *L* Lubrication, *O* Orgasm, *VP* No problems with vaginal penetration, *AA* Absence of anticipatory anxiety, *SI* Sexual initiative, *SC* Degree of sexual communication, *SA* Satisfaction with sexual activity, *SS* Overall sexual satisfaction, *FS* Frequency of sexual activity

Lastly, the multivariable model for resumption of sexual activity postpartum (Table 4) included the frequency of sexual activity ‘and’ second-degree tears as predictors, accounting for approximately 16.3% of the variance in the initiation of sexual intercourse (*R*^2^ = 0.16). The model was statistically significant (*F* = 6.41, *p* = 0.00). Specifically, the occurrence of a second-degree tear instead of episiotomy was associated with a decrease in the initiation time of sexual intercourse (*B* = −10.45, *p* = 0.01), and a higher frequency of sexual activity also predicted a reduction in the initiation time (*B* = −5.00, *p* = 0.022).

**Table 4:** Results of stepwise multiple regression analysis for the resumption of sexual activity postpartum

	*B*	SD	Beta	*t*	*p*-value
Second-degree tear vs. episiotomy	−10.45	4.09	−.28	−2.55	.01
Frequency of Sexual Activity	−5.00	2.13	−.26	−2.34	.02

Adjusted *R*^2^ = .13; *F* = 6.41; *p* = .00

## Discussion

This study examined the association between second-degree tears and episiotomy during childbirth, and variables related to sexual health. This study focused on second-degree and episiotomy tears. Moreover, the literature suggests that episiotomy increases the likelihood of dyspareunia and impaired sexual function at 3 months postpartum compared with intact perineum or first-degree tears [7, 24]. However, this study compared women who underwent episiotomies with those who had second-degree tears and found no major differences in dyspareunia and sexual function. The significance of the predictors in our multivariate model, including the frequency of sexual activity and the presence of second-degree tears, highlights their relevance in the resumption of sexual intercourse after childbirth. These factors explain a substantial proportion of the observed variance.

Notably, women who underwent episiotomies waited 1–2 weeks longer to resume sexual intercourse than did the other groups, which is consistent with the literature^5^. Statistical analysis demonstrated a significant relationship between the resumption of postpartum sexual activity and the occurrence of second-degree perineal tears, in contrast to episiotomy. Various psychological elements, including concerns about pain, self-esteem, and relationship dynamics contribute to the delayed resumption of sexual activity. On the other hand, physical factors, notably healing and hormonal fluctuations, also influence the resumption of postpartum sexual activity. Sociocultural factors such as cultural norms and communication within the partnership also play a role in postpartum sexual activity. Our study supports the notion that increased sexual activity is associated with faster resumption of sexual relations following childbirth, as reflected by the negative coefficients in our regression analysis. The multivariate model also revealed a clear inverse relationship between the frequency of sexual activity and time taken to initiate intercourse. Specifically, the model showed that a higher frequency of sexual activity was associated with a shorter time to resume sexual activity postpartum. This finding aligns with the literature, which suggests that sexual health recovery is a multifaceted process influenced not only by physical healing but also by psychological readiness and confidence in resuming sexual relations [25, 26]. The negative coefficient for sexual activity frequency points to the potential role of pre-existing sexual habits in facilitating a quicker return to sexual activity, possibly due to maintained physiological conditions conducive to sexual function or more robust sexual communication between partners that persists through the postpartum period.

Interestingly, age and parity were associated with perineal trauma (usually tears), rather than episiotomy. Corroborating this finding, other studies have shown that multiparous women have more tears than primiparous women [27]. One possible explanation is that multiparous women’s birth process is faster; therefore, fewer interventions, such as episiotomy, are needed. Furthermore, age was associated with more births and tears than episiotomy was. In terms of sexual dynamics, the study sample had moderate sexual function disorder. Consistent with this, the literature shows that women initiate sexual activity 54% of the time, and 45% and 1% of the time before, during, and after pregnancy, respectively. The predictive capacity of our model, despite accounting for only 16.3% of the variance, indicated a statistically significant association between the identified predictors and the initiation of sexual intercourse postpartum at an early stage.

Higher education was associated with later onset and lower frequency of postpartum sexual activity. This could be attributed to employment being linked to the later onset of sexual relations after childbirth. Maternal roles, especially when combined with other responsibilities such as work or studies, might lead to the neglect of personal needs, including sexual activity, due to time constraints and physical and emotional conditions [27]. Similarly, a higher educational level was associated with a lower frequency of sexual activity during breastfeeding. This aligns with the idea that higher maternal education is linked to longer breastfeeding duration, likely because of greater awareness of its benefits and training. Despite existing beliefs, this study did not find a higher frequency of dyspareunia in breastfeeding women, even though the literature suggests a link between dyspareunia and hormonal changes [28]. In a parallel study, the authors linked dyspareunia and breastfeeding 12 weeks after birth, but not to perineal trauma [7]. However, no significant relationship was observed in this study. Additionally, this study found that reduced dyspareunia was associated with desire, arousal, orgasm, lack of anticipatory anxiety, sexual initiative, lack of penetration, and satisfaction with sex. As highlighted in recent research, the literature states that dyspareunia reduces sexual desire in women [29]. Sexual desire is related to other highly correlated sexual health dimensions. For example, desire and arousal have been linked to lubrication, orgasm, and sexual satisfaction. In a related study, orgasm, satisfaction, anticipatory anxiety, and penetration were strongly correlated. This finding implies that dyspareunia can indirectly affect these dimensions. It is crucial to emphasize that dyspareunia is a painful and unpleasant experience that makes sex less enjoyable and can be avoided. On the brighter side, good sexual health, such as high desire, arousal, or orgasm, may protect against dyspareunia as suggested by this correlation. To achieve a fulfilling sexual relationship, intimacy is essential for sexual function, which requires a woman to respond to her partner’s sexual stimuli or to feel desire spontaneously. Considering this broader picture, several post-birth factors must be considered to achieve this goal. For instance, they include the home environment, which is often disrupted by the needs of newborns. This shift has led to a new family model that focuses on newborns, which can change the expression of emotions between spouses and the time devoted to couples. Moreover, a positive self-image, which can be affected by physical and psychological changes in the puerperium, is necessary to foster trust and intimacy. Consequently, if sexual stimuli meet expectations, biological and psychological factors will lead to desire, arousal, and orgasm, which will provide women with physical and emotional satisfaction and allow them to view sexuality in a healthy manner [30]. The outcomes of this study demonstrate the crucial elements that could have an impact on clinical practices and recommendations for postpartum care. The coefficients obtained for second-degree tears and the frequency of sexual activity in our model are noteworthy findings that could shape the development of postpartum care guidelines.

This study has some limitations. First, random sampling was not employed, limiting the generalizability of the findings. Second, due to the small sample size, robust statistical analyses could not be conducted. Self-reported measures were utilized, which may have been influenced by factors such as social desirability and expectation bias, and the study’s design only assessed participants’ perceptions at one-time point, preventing the tracking of changes over time. Certain conditions and characteristics relevant to women were excluded from the analysis, potentially leading to the loss of important factors. The study’s use of telephone or electronic data collection methods may introduce bias due to the lack of face-to-face interactions and their impact on response accuracy and precision. Additionally, the study’s cross-sectional design prevented the establishment of causal relationships between variables. The lack of sufficient baseline studies posed challenges in determining the optimal sample size, potentially affecting the representativeness of the participants. Finally, while we followed the STROBE guidelines diligently, there may be variability in their interpretation and application, which could influence both the methodology and the results obtained.

## Conclusions

This study aimed to explore the relationship between perineal trauma and postpartum sexual health. It was found that factors such as psychological, physical, and sociocultural influences affect women’s ability to resume sexual activity after childbirth. This study suggests that midwives and healthcare professionals should consider these factors when providing support to postpartum women. These findings can be used to improve the care of women during the postpartum period.

## Data Availability

The data supporting the results of this study are available from the corresponding author upon request, with access to details and conditions provided upon direct contact.

## Declarations

### Relevance to clinical practice

In the future, this study’s findings can be used to formulate hypotheses that address its limitations. For example, further research could include a control group of women without tears or episiotomy, and expand the sample size. It is apparent that couples may experience anxiety when resuming sexual activity following episiotomy or tearing. Therefore, it is important for midwives to evaluate sexual function during postpartum care to assist couples. Midwives who assess postpartum sexual function should prioritize several key aspects. These include specialized training in sexual health, creating a confidential and comfortable environment for discussion, obtaining informed consent from women, using validated questionnaires to assess sexual function, initiating open-ended discussions to encourage participation, maintaining a non-judgmental approach throughout the process, addressing prevalent concerns sensitively and effectively, and providing educational resources to support women’s understanding of their own sexuality after childbirth. In addition, it is essential for midwives to develop a standardized process for referring patients to specialized care when needed and arrange personalized follow-up appointments. It is also important for midwives to approach postpartum sexuality with cultural sensitivity when working with diverse populations facing challenges in this area. Thorough documentation of all interactions should be maintained while ensuring the privacy and confidentiality of the women. Providing comprehensive care includes addressing sexual health concerns, and midwives can utilize research findings to provide appropriate guidance on resuming sexual activity after various types of perineal injuries, considering individual recovery times. Informing women about the anticipated timeline for resuming sexual intercourse, specifically in relation to spontaneous tears and episiotomies, can assist them in establishing realistic expectations and reducing their anxiety. In light of this, midwives can tailor postpartum care plans based on whether a woman has experienced a tear or has undergone an episiotomy. For example, additional check-ups or supplementary resources may be offered to women who have undergone an episiotomy, as they may require more time before feeling comfortable engaging in sexual activity again. Emphasizing the emotional dimension, this study highlights the importance of providing emotional support during the postpartum period. Midwives have the opportunity to collaborate with psychologists and therapists to provide emotional support for women experiencing difficulties in postpartum sexual recovery. By facilitating open dialogue, midwives can encourage women to have honest conversations with their partners regarding their fears, expectations, and boundaries regarding sexual activity after childbirth. This collaborative approach has the potential to enhance couples’ relationships and help them manage their expectations. Furthermore, these findings may provide valuable insights for future research on interventions aimed at assisting women in resuming sexual activities comfortably and efficiently. In clinical practice, midwives should consider individualized interventions based on educational background, marital status, and breastfeeding status. Recognizing that the process of sexual recovery is dynamic in nature, it is important for midwives to routinely identify emerging issues and address them accordingly.

## References

[1] Trento M, Charrier L, Salassa M, Merlo S, Passera P, Cavallo F, Porta M (2021). A cross-sectional survey on impact of the COVID-19 lockdown on glycaemic control, physical activity, and mood in people with type 1 diabetes: the 3LOCK survey. Acta Diabetol.

[2] Costa CLD, Spyrides MHC, Sousa MDLRD (2018) Consistency of three different questionnaires for evaluating sexual function in healthy young women. BMC Women’s Health 18(1):189 10.1186/s12905-018-0693-y PMC6302409 30572853

[3] Rezaei N, Azadi A, Sayehmiri K, Valizadeh R, Tavan H (2018). Postpartum sexual functioning and its predicting factors among Iranian women. Malays J Med Sci.

[4] Woretaw AZ, Tessema GA, Alemu BM (2021). Postpartum sexual activity and associated factors among women who gave birth in the last 12 months in Debre Markos town, Northwest Ethiopia. BMC Women’s Health.

[5] Nyaloko M, Lubbe W, Minnie K (2022) An integrative literature review on factors affecting breastfeeding in public spaces. Open Public Health J 15(1)

[6] O'Malley D, Higgins A, Begley C, Daly D, Smith V (2018). Prevalence of and risk factors associated with sexual health issues in primiparous women at 6 and 12 months postpartum; a longitudinal prospective cohort study (the MAMMI study). BMC Pregnancy Childbirth.

[7] Hidalgo-Lopezosa P, Pérez-Marín S, Jiménez-Ruz A, López-Carrasco JC, Cubero-Luna AM, García-Fernández R, Rodríguez-Borrego MA, Liébana-Presa C, López-Soto PJ (2022). Factors associated with postpartum sexual dysfunction in Spanish women: a cross-sectional study. J Personalized Med.

[8] Edosa H, Tefera Y, Mulatu T (2022). Prevalence and associated factors of postpartum sexual dysfunction among women who gave birth in the last 12 months in Aneded district, Northwest Ethiopia. BMC Women’s Health.

[9] Shinohara H, Suganuma N, Ito K, Kanou H, Takiguchi S, Tanaka K (2017). Relationship between sexual distress and psychological distress during the first year postpartum: a prospective cohort study. Sex Med.

[10] Natalie D, Frawley H (2022). The relationship between childbirth and symptoms of pelvic floor disorder: a literature review. Women Birth.

[11] Mojdeh B, Fatemeh A (2021). The effect of pelvic floor muscle training on sexual function in postpartum women: a randomised controlled trial. J Caring Sci.

[12] Naglaa MA, El-Sayed N (2023). Postpartum sexual function and associated factors: an observational cross-sectional study. Sex Med.

[13] Deirdre J, O’Malley D (2018). Postpartum sexual health: a principle-based concept analysis. J Adv Nurs.

[14] Raaberg M, Glavind J, Møller LK (2022). Sexual function in the postpartum period after first-time mediolateral episiotomy: a prospective matched case-control study. Sex Med.

[15] Gudushauri P, Whitcomb BW, Louis GM (2023). Sexual function in women with and without a history of infertility. Sex Med.

[16] Ahmed AS, Sayed M, Mohammed H (2017). Postpartum sexual function and its predicting factors among Egyptian women. Sex Med.

[17] Raut V, Mutiso S (2019). Postpartum sexual function in women and the effects of early resumption of intercourse. Sex Med.

[18] Padoa A, Rosenbaum TY (2023). The sexual health of women after pelvic surgery. Sex Med.

[19] Sharp MK, Bertizzolo L, Rius R, Wager E, Gómez G, Hren D (2019). Using the STROBE statement: survey findings emphasized the role of journals in enforcing reporting guidelines. J Clin Epidemiol.

[20] Sánchez F, Conchillo MP, Valls JB, Llorens OG, Vicentee JA, de Las Mulas ACM (2004). Diseño y validación del cuestionario de Función Sexual de la Mujer (FSM). Atención primaria.

[21] Chander NG (2019). Visual analog scale in prosthodontics. J Indian Prosthodont Soc.

[22] Kelley K, Preacher KJ (2012). On effect size. Psychol Methods.

[23] Gommesen D, Nøhr E, Qvist N, Rasch V (2019). Obstetric perineal tears, sexual function and dyspareunia among primiparous women 12 months postpartum: a prospective cohort study. BMJ Open.

[24] Alum AC, Kizza IB, Osingada CP, Katende G, Kaye DK (2015). Factors associated with early resumption of sexual intercourse among postnatal women in Uganda. Reprod Health.

[25] Delgado-Pérez E, Rodríguez-Costa I, Vergara-Pérez F, Blanco-Morales M, Torres-Lacomba M (2022) Recovering sexuality after childbirth. What strategies do women adopt? A qualitative study. Int J Environ Res Public Health 19(2):950 10.3390/ijerph19020950 PMC8775547 35055771

[26] Adler K, Rahkonen L, Kruit H (2020). Maternal childbirth experience in induced and spontaneous labour measured in a visual analog scale and the factors influencing it; a two-year cohort study. BMC Pregnancy Childbirth.

[27] Ahmadifaraz M, Foroughipour A, Abedi H, Azarbarzin M, Dehghani L, Meamar R (2013). Anxiety of women employees and the process of maternal role. Int J Prev Med.

[28] Krysiak R, Szkróbka W, Okopień B (2018). The effect of bromocriptine treatment on sexual functioning and depressive symptoms in women with mild hyperprolactinemia. Pharmacol Rep.

[29] Romashchenko O, Grygorenko V, Biloholovska V, Babych O, Melnykov S, Yakovenko L (2022). Use of platelet-rich plasma for the treatment of dyspareunia in postmenopausal women. J Sex Med.

[30] Lara LAS, Scalco SCP, Rufino AC, Paula SRC, Fernandes ES, Pereira JML, França SS, Reis S, Almeida SB, Vale FBC, Lerner T, Carvalho YMV, Abdo CHN, Oliveira FFL (2021). Management of hypoactive sexual desire disorder in women in the gynaecological setting. Rev Bras Ginecol Obstet.

